# Association of plasma apolipoprotein CIII, high sensitivity C-reactive protein and tumor necrosis factor-α contributes to the clinical features of coronary heart disease in Li and Han ethnic groups in China

**DOI:** 10.1186/s12944-018-0830-5

**Published:** 2018-07-27

**Authors:** Lin Chen, Minzeng Sun, Hui Liu, Lihui Ma, Tiansong Wang, Peiqiong Li, Mingqin Lin, Haifeng Lin, Penghuan Chang, Yueli Liu

**Affiliations:** 1 Department of Pharmacology, School of Basic Medicine and Life Sciences, Hainan Medical University, Haikou, 571199 Hainan Province China; 2 Department of Cardiology, People’s Hospital of Sanya, Sanya, 572000 Hainan Province China; 3 Department of Anatomy, School of Basic Medicine and Life Sciences, Hainan Medical University, Haikou, 571199 Hainan Province China; 4 Department of Oncology, The Second Affiliated Hospital of Hainan Medical University, Haikou, 570311 Hainan Province China; 5 Department of Gynecology and Obstetrics, Haikou people’s Hospital, Haikou, 570208 Hainan Province China

**Keywords:** ApoCIII, Hs-CRP, TNF-α, Association, Coronary artery disease

## Abstract

**Background:**

Apolipoprotein CIII (apoCIII) is an independent risk for coronary heart disease (CHD). In this study, we investigated the associations among plasma apoCIII, hs-CRP and TNF-α levels and their roles in the clinical features of CHD in the Li and Han ethnic groups in China.

**Methods:**

A cohort of 474 participants was recruited (238 atherosclerotic patients and 236 healthy controls) from the Li and Han ethnic groups. Blood samples were obtained to evaluate apoCIII, TNF-α, hs-CRP and lipid profiles. Chi-squared, *t*-tests, and Kruskal–Wallis or Wilcoxon–Mann–Whitney tests, Pearson or Spearman correlation tests and multiple unconditional logistic regression were employed to analyze lipid profiles and variations in plasma apoCIII, TNF-α, hs-CRP in subgroups of CHD and their contributions to CHD using SPSS version 20.0 software.

**Results:**

Compared to healthy participants, unfavorable lipid profiles were identified in CHD patients with enhanced systolic pressure, diastolic pressure, fasting blood sugar (FBS), TG, TC, LDL-C, apoB, Lp(a) (*P* < 0.05, TC and Lp(a); *P* < 0.01, FBS, TG, LDL-C, apoB); and lower HDL-C and apoAI (*P* < 0.05). Plasma apoCIII, TNF-α and hs-CRP levels were higher in CHD individuals (16.77 ± 5.98 mg/dL vs. 10.91 ± 4.97 mg/dL; 17.23 ± 6.34 pg/mL vs. 9.49 ± 3.88 pg/mL; 9.55 ± 7.32 mg/L vs. 2.14 ± 1.56 mg/L; *P* < 0.01 vs. healthy participants). Identical patterns were obtained in the Li and Han groups (16.46 ± 6.08 mg/dL vs. 11.72 ± 5.16 mg/dL; 15.71 ± 5.52 pg/mL vs. 9.74 ± 4.31 pg/mL; 8.21 ± 7.09 mg/L vs. 2.15 ± 1.51 mg/L in Li people; 17.05 ± 5.90 mg/dL vs. 10.07 ± 4.63 mg/dL; 18.59 ± 6.73 pg/mL vs. 9.23 ± 3.38 pg/mL; 10.75 ± 7.44 mg/L vs. 2.12 ± 1.63 mg/L in Han people; *P* < 0.01). Paired comparisons of subgroups with stable angina, unstable angina, and acute myocardial infarction (AMI) revealed significant variation in plasma levels of apoCIII, TNF-α and hs-CRP (*P* < 0.01), but not among subgroups with mild, moderate and severe stenosis (*P* > 0.05). Plasma apoCIII, TNF-α and hs-CRP contributed to the development of CHD (OR = 2.554, 7.252, 6.035, *P* < 0.01) with paired correlations in CHD patients (apoCIII vs. TNF-α, *r* = 0.425; apoCIII vs. hs-CRP, *r* = 0.319; TNF-α vs. hs-CRP, *r* = 0.400, *P* < 0.01).

**Conclusions:**

Association among plasma apoCIII, hs-CRP and TNF-α interacts with unfavorable lipid profiles to contribute to the clinical features of CHD with stable angina, unstable angina, and AMI in the Li and Han ethnic groups in China.

## Background

Coronary heart disease (CHD) is characterized by dyslipedemia and chronic inflammation. Many studies have shown that apolipoprotein CIII (apoCIII) is an independent risk factor for CHD and promotes adhesion of inflammatory mediators to endothelial cells, which aggravates the progression of atherosclerosis [1–6]. ApoCIII is reported to be associated with lipoprotein-associated phospholipase A2 (Lp-PLA_2_), which catalyzes the hydrolysis of oxidized low density lipoprotein (ox-LDL) and the release of inflammatory products and is also found in ruptured plaques of human atherosclerotic lesions [7–9]. Therefore, it is postulated that apoCIII is involved in the formation and instability of atherosclerotic plaques. Plasma apoCIII is reported to be associated with high-sensitivity C-reactive protein (hs-CRP) and tumor necrosis factor α (TNF-α) levels [10]. However, the relationships between plasma apoCIII, hs-CRP and TNF-α in patients with CHD and their roles in coronary artery narrowing as well as the clinical features of CHD remain to be elucidated. In this study, we investigated the associations among plasma apoCIII, hs-CRP and TNF-α levels and their roles in the clinical features of CHD in the Li and Han ethnic groups in China.

## Methods

### Study population

**Fig. 1:**
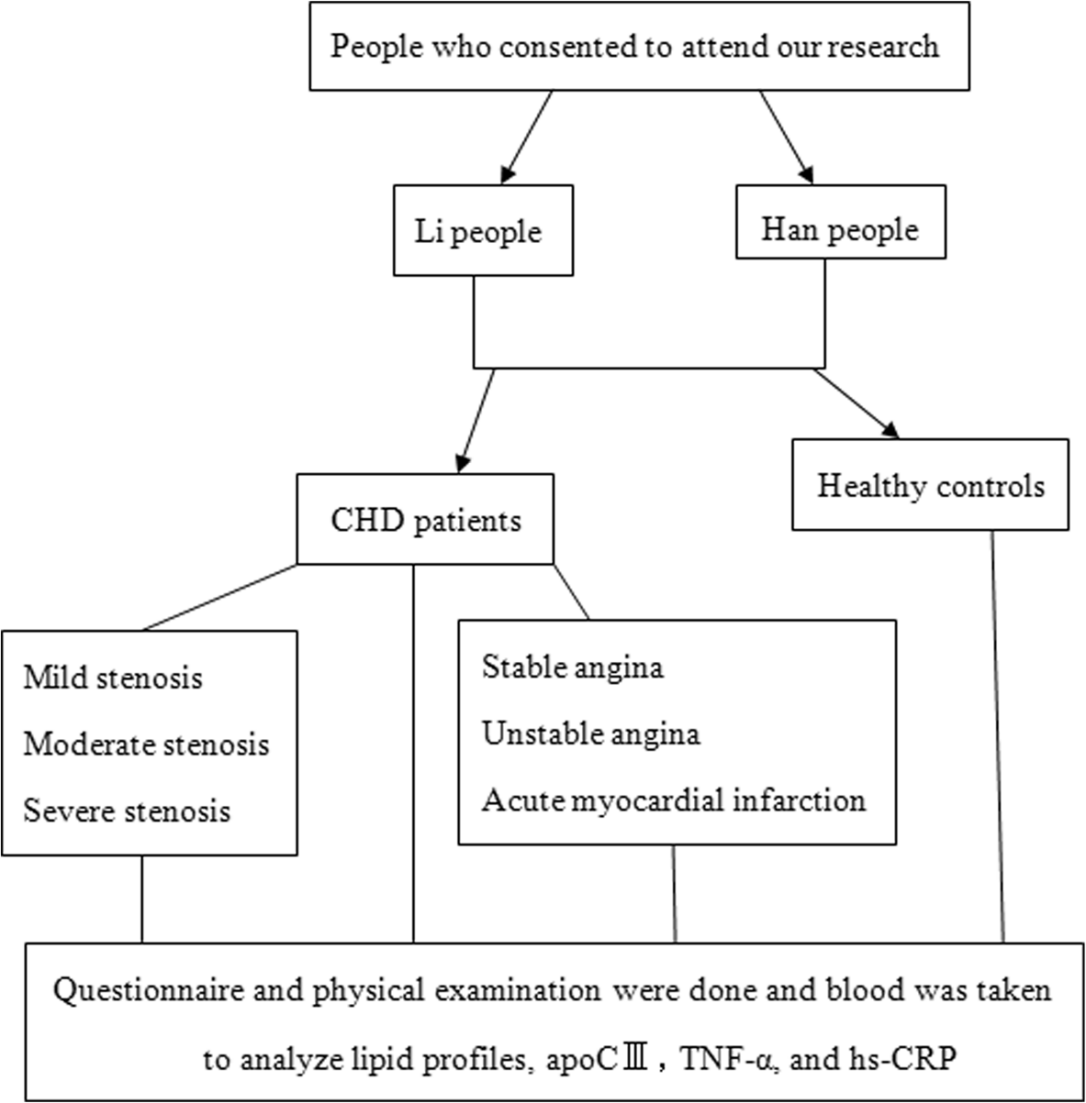
Flow chart of our study protocol

Our study was approved by the Ethics Committee of the First Affiliated Hospital of Hainan Medical University. Written informed consent was obtained from all participants. This study was performed following the rules of the Declaration of Helsinki. A flow chart of our study protocol was shown in Fig. 1. From September 2012 to November 2014, a total of 474 participants were recruited. This group consisted of 238 CHD patients (CHD group) comprising 112 Li individuals and 126 Han individuals (average age 62.56 ± 12.34 y), and 236 healthy participants (control group) comprising 120 Li individuals and 116 Han individuals (average age 60.76 ± 11.04 y). All participants were aged between 38 and 84 y. There was no consanguinity among the participants, whose grandparents and parents were from the same population and had resided in Hainan Province for more than 10 years.

CHD was defined as angiographically proven stenosis (> 50%) of at least one major epicardial coronary artery. Patients in the CHD group were subdivided into stable angina, unstable angina, and acute myocardial infarction (AMI) groups according to the clinical features of CHD. Stable angina was confirmed according to the diagnostic criteria for CHD (WS 319–2010) released by the Ministry of Health of China; unstable angina and AMI were confirmed according to the third universal definition of AMI and advanced cardiology [11]. Measurement of cardiac enzyme activity [creatine kinase(CK), CK- MB isoenzyme, cardiac troponin I (CTNI), myoglobin (MYO)] and electrocardiography (ECG) were used to confirm stable angina, unstable angina, and AMI. ECG measurements were conducted in 12-lead examinations, or if required, 18-lead examinations as a replacement. ECG results were interpreted by experienced clinicians.

AMI diagnosis was based on the detection of increased and/or decreased cardiac biomarkers (preferably troponin) including at least one value above the 99th percentile of the upper reference limit together with evidence of myocardial ischemia with at least one of the following: (1) Symptoms of ischemia; (2) ECG changes indicative of new ischemia; (3) Development of pathological Q-waves in the ECG; (4) Imaging evidence of new loss of myocardium or new regional wall motion abnormality; (5) Coronary thrombus confirmed by coronary angiography.

Unstable angina was diagnosed under at least one of the following conditions: (1) Diagnosed stable angina and increasing frequency, duration and severity of chest pain within the previous 2 months; (2) Angina attack at rest or during mild physical activity; (3) Temporarily depressed ST segment in ECG with/without T-wave inversion; (4) Normal or slightly increased cardiac biomarkers; (5) Coronary heart disease confirmed by coronary angiography.

Stable angina was diagnosed under at least one of the following conditions: (1) Typical symptoms of angina attack; (2) Angina staying stable within the previous 1–3 months; (3) Ischemic ST dynamic change in ECG during angina attack or positive exercise tolerance test; (4) Confirmed by coronary angiography or CT angiography.

CHD patients were also subdivided into mild stenosis, moderate stenosis and severe stenosis subgroups according to results of angiography scored on the basis of the revised Gensini scoring system [12]; details are shown below.

CHD patients were screened on the basis of having no history of chronic or acute cerebrovascular disease, inflammatory disease, autoimmune disease, malignant tumors, familial hyperlipidemia, diabetes or other diseases or incomplete data which affected the results of our study. Healthy participants were screened on the basis of having no history of hypertension, cardiovascular and cerebrovascular disease, endocrinal disease, malignant tumors, genetic disease, transmitted diseases, renal and hepatic dysfunction or other diseases or incomplete data affecting the results of our study. CHD patients and healthy controls were also excluded if they were maintained on anti-hyperlipidemic agents, anti-inflammatory agents, nutraceuticals and functional foods or other agents known to affect lipid and inflammatory profiles in the previous 3 months.

### Epidemiological survey

Demographic information was collected and recorded using a standardized questionnaire. With the exception of general information such as name, sex and age, smoking and alcohol status were recorded as two groups: non-smoker and smoker, and non-drinker and drinker. Blood pressure referred to the average sitting blood pressure after three measurements made using a mercurial sphygmomanometer. Systolic and diastolic blood pressures referred to the first and the fifth Korotkoff sounds, respectively.

#### Biochemical analysis

Fasting blood samples were obtained to analyze plasma triglyceride (TG), total cholesterol (TC), low density lipoprotein cholesterol (LDL-C), high density lipoprotein cholesterol (HDL-C), lipoprotein AI (apoAI), lipoprotein B (apoB), apoCIII, lipoprotein a (Lp(a)), fasting blood sugar (FBS), hs-CRP, and TNF-α. TG, TC, HDL-C, LDL-C, and FBS were assayed using enzymatic methods with commercially available kits. ApoAI, apoB, Lp(a), apoCIII and TNF-α levels were measured by enzyme-linked immunosorbent assay (ELISA). Hs-CRP was measured by latex turbidity test. All samples were processed by autoanalyzer (Type 7600; Hitachi Ltd., Tokyo, Japan) or ELISA reader (Biotech, USA) at the First Affiliated Hospital of Hainan Medical University.

### Evaluation of coronary artery stenosis

Coronary artery stenosis examined by percutaneous coronary angiography was classified according to revised Gensini scoring system. Six branches of the coronary artery (left main coronary artery, left anterior descending artery, left circumflex branch, left obtuse marginal branch, right coronary artery, and posterior descending artery) were examined and scored according to the narrowest section of the branches. The scores were assigned as follows: 0, without narrowing; 1, 1–49% narrowing; 2, 50–74% narrowing; 3, 75–99% narrowing; 4, 100% narrowing. The total score was determined as the sum of the score of the six branches of the coronary artery. The total scores were defined as follows: 1–7, mild stenosis; 7–14, moderate stenosis; >14, severe stenosis.

### Statistical analysis

Statistical analysis was carried out using SPSS version 20.0 software, and data were presented as the mean ± standard deviation (SD). Quantitative values were analyzed by *t-* test; counting data was analyzed by the chi-squared test. Comparisons of intergroup differences in apoCIII, hs-CRP and TNF-α levels in patients with CHD were analyzed using Kruskal–Wallis or Wilcoxon–Mann–Whitney tests. The contribution of plasma apoCIII, hs-CRP and TNF-α to CHD were analyzed using multiple unconditional logistic regression. Paired associations among apoCIII, hs-CRP and TNF-α in CHD patients were analyzed using the Pearson or Spearman correlation test. *P-*values < 0.05 were considered to indicate statistical significance.

## Results

### Comparison of general demographic features and lipid profiles between CHD patients and healthy controls

There were no significant differences between the CHD patients and healthy controls included in the study in terms of the Li:Han ratio, male:female ratio, mean age, smoking status and drinking status (all *P* > 0.05). CHD patients exhibited higher systolic pressure, diastolic pressure, FBS, TG, TC, LDL-C, apoB, and Lp(a) than healthy controls (*P* < 0.05 for TC and Lp(a); *P* < 0.01 for FBS, TG, LDL-C and apoB), while HDL-C and apoAI were lower (*P* < 0.05 for both) (Table 1).

**Table 1:** Comparison of general demographic features and lipid profiles between patients with coronary heart disease (CHD) and healthy controls (control)

Characteristic	CHD (*n* = 238)	Control (*n* = 236)	*t* (*χ*^*2*^)	*P-*value
Nationality (Li/Han)	112/126	120/116	0.681	0.409
Sex (male/female)	138/100	119/117	2.728	0.099
Age (years)	62.56 ± 12.34	60.76 ± 11.04	1.674	0.095
Systolic pressure (mmHg)	134.07 ± 21.54^**^	128.50 ± 14.61	29.204	0.000
Diastolic pressure (mmHg)	78.94 ± 11.71^**^	77.56 ± 7.87	13.18	0.000
Non-smoker/smoker	77/161	59/177	3.131	0.077
Non-drinker/drinker	46/192	63/173	3.632	0.057
FBS (mmol/L)	5.92 ± 1.51^**^	5.65 ± 0.47	2.66	0.008
TC (mmol/L)	4.95 ± 0.96^*^	4.79 ± 0.75	2.024	0.044
TG (mmol/L)	1.47 ± 1.05^**^	1.18 ± 0.73	3.542	0.000
HDL-C (mmol/L)	1.06 ± 0.34^**^	1.28 ± 0.30	−7.441	0.000
LDL-C (mmol/L)	3.08 ± 0.83^**^	2.82 ± 0.72	3.709	0.000
ApoAI (g/L)	1.16 ± 0.36^**^	1.32 ± 0.35	−4.936	0.000
ApoB (g/L)	1.04 ± 0.24^**^	0.87 ± 0.17	8.732	0.000
Lp(a) (mg/L)	257.41 ± 189.07^*^	217.44 ± 165.27	2.451	0.015

Data are expressed as mean ± SD. *FBS* Fasting blood sugar, *TC* Total cholesterol, *TG* Triglyceride, *HDL-C* High density lipoprotein cholesterol, *LDL-C* Low density lipoprotein cholesterol, *ApoAI* Lipoprotein AI, *ApoB* Lipoprotein B; *Lp(a)* Lipoprotein a, *CHD* Coronary heart disease. ^*^P < 0.05, ^**^P < 0.01, CHD patients versus healthy controls using *χ*^*2*^ test or *t-*test

### Comparison of plasma apoCIII, hs-CRP and TNF-α levels between CHD patients and healthy controls in the li and Han ethnic groups

Plasma apoCIII, hs-CRP and TNF-α levels in CHD patients were higher than those in the healthy controls (*P* < 0.01 for all) (Table 2). Identical patterns were obtained in further comparisons between CHD patients and healthy controls in the Li and Han ethnic groups (*P* < 0.01) (Table 3). In addition, in the Li group, we observed enhanced hs-CRP and TNF-α levels in CHD patients and a higher apoCIII level in healthy controls compared to the levels detected in the Han group (*P* < 0.01 for hs-CRP and TNF-α; *P* < 0.05 for apoCIII) (Table 4).

**Table 2:** Comparison of plasma apoCIII, TNF-α and hs-CRP between patients with coronary heart disease (CHD) and healthy controls (control)

Parameter	CHD (*n* = 238)	Control (*n* = 236)	*t*	*P*-value
ApoCIII (mg/dL)	16.77 ± 5.98**	10.91 ± 4.97	11.606	0.000
TNF-α (pg/mL)	17.23 ± 6.34**	9.49 ± 3.88	16.043	0.000
Hs-CRP (mg/L)	9.55 ± 7.32**	2.14 ± 1.56	15.176	0.000

Data are expressed as mean ± SD. *ApoCIII* Apolipoprotein CIII, *TNF-α* Tumor necrosis factor α, *Hs-CRP* High-sensitivity C-reactive protein, *CHD* Coronary heart disease. ^**^P < 0.01, CHD patients versus healthy controls using *t-*test

**Table 3:** Comparison of plasma apoCIII, TNF-α and hs-CRP levels between patients with coronary heart disease (CHD) and healthy controls (control) in the Li and Han ethnic groups

Parameter	Li ethnic group			Han ethnic group		
	CHD (*n* = 112)	Control (*n* = 120)	*P-*value	CHD (*n* = 126)	Control (*n* = 116)	*P*-value
ApoCIII (mg/dl)	16.46 ± 6.08**	11.72 ± 5.16	0.000	17.05 ± 5.90**	10.07 ± 4.63	0.000
TNF-α (pg/ml)	15.71 ± 5.52**	9.74 ± 4.31	0.000	18.59 ± 6.73**	9.23 ± 3.38	0.000
Hs-CRP (mg/l)	8.21 ± 7.09**	2.15 ± 1.51	0.000	10.75 ± 7.44**	2.12 ± 1.63	0.000

Data are expressed as mean ± SD. *ApoCIII* Apolipoprotein CIII, *TNF-α* Tumor necrosis factor α, *Hs-CRP* high sensitive C-reactive protein, *CHD*: coronary heart disease. ^**^P < 0.01, CHD patients versus healthy controls using *t*-test

**Table 4:** Comparison of plasma apoCIII, TNF-α and hs-CRP levels between Li and Han people in coronary heart disease patients (CHD) and healthy controls (control)

Parameter	CHD			Control		
	Li people (*n* = 112)	Han people (*n* = 126)	*P*-value	Li people (*n* = 120)	Han people (*n* = 116)	*P*-value
ApoCIII (mg/dl)	16.46 ± 6.08	17.05 ± 5.90	0.448	11.72 ± 5.16*	10.07 ± 4.63	0.011
TNF-α (pg/ml)	15.71 ± 5.52**	18.59 ± 6.73	0.000	9.74 ± 4.31	9.23 ± 3.38	0.314
Hs-CRP (mg/l)	8.21 ± 7.09**	10.75 ± 7.44	0.008	2.15 ± 1.51	2.12 ± 1.63	0.908

Data are expressed as mean ± SD. *ApoCIII* Apolipoprotein CIII, *TNF-α* Tumor necrosis factor α, *Hs-CRP* High-sensitivity C-reactive protein, *CHD* Coronary heart disease.^*^P < 0.05, ^**^P < 0.01, Li people versus Han people in CHD and control groups using *t*-test

### Intergroup comparison of plasma apoCIII, hs-CRP and TNF-α levels in CHD patients

**Fig. 2:**
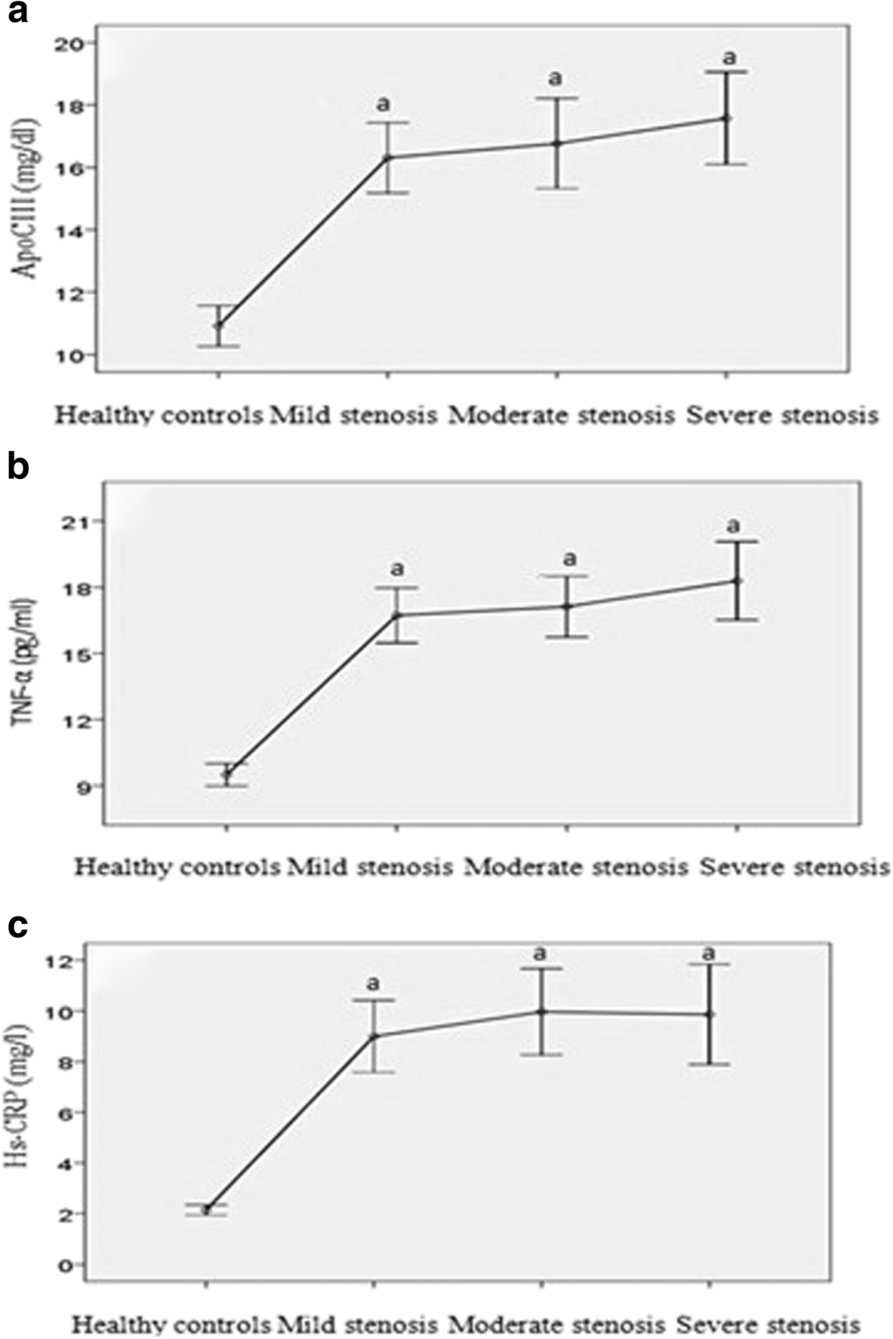
Paired comparisons of plasma apoCIII, TNF-α and hs-CRP levels in subgroups with severe, moderate, and mild stenosis and healthy controls (control). **a**: Paired comparisons of plasma apoCIII levels in subgroups with severe, moderate, and mild stenosis, and control; **b**: Paired comparisons of plasma TNF-α levels in subgroups with severe, moderate, and mild stenosis, and control; **c**: Paired comparisons of plasma hs-CRP levels in subgroups with severe, moderate, and mild stenosis, and control. a: compared to control, *P* < 0.01; there were no significant differences between each pair of subgroups with severe, moderate and mild stenosis (*P* > 0.05)

**Fig. 3:**
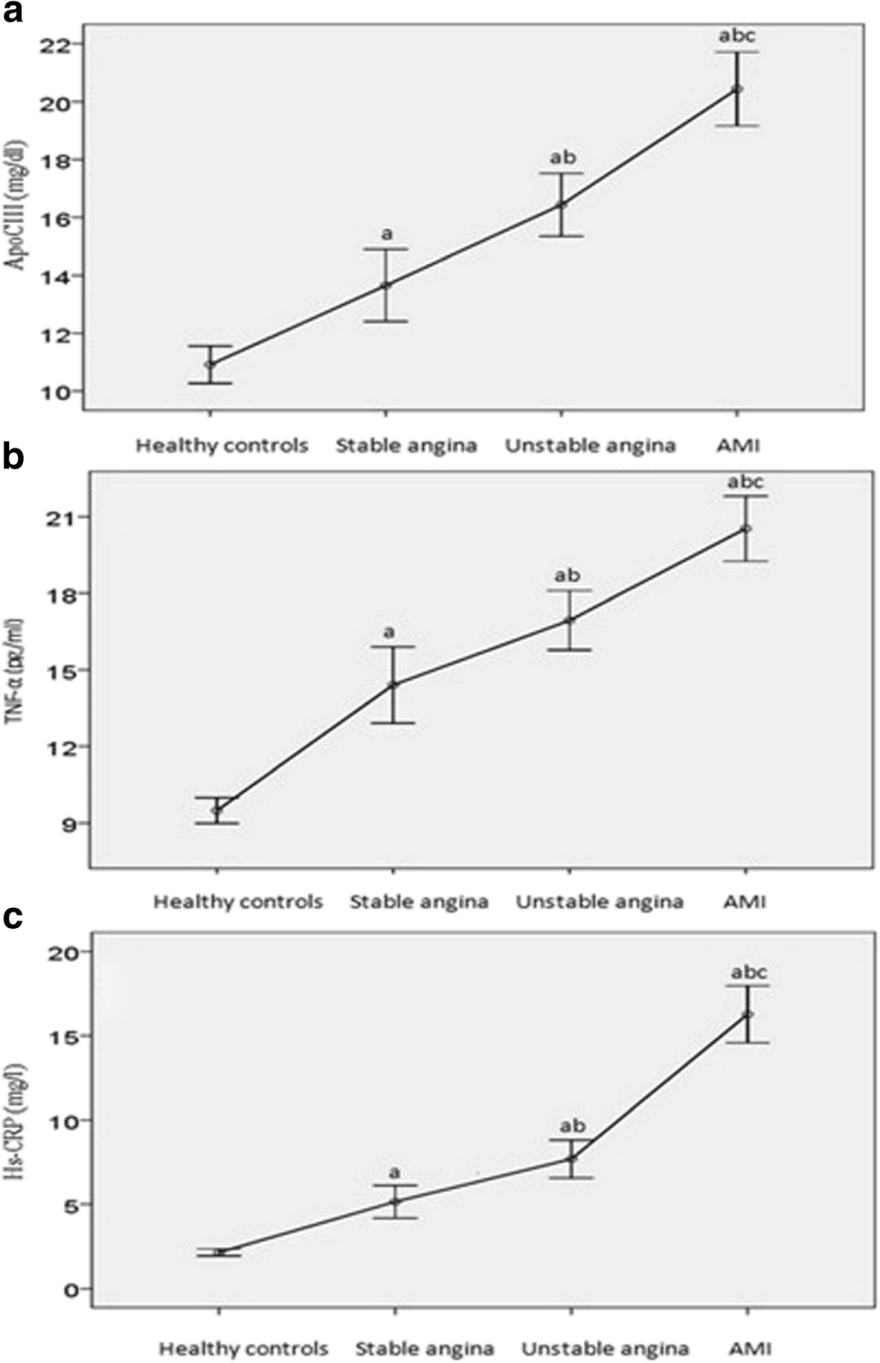
Paired comparisons of plasma apoCIII, TNF-α and hs-CRP levels in subgroups with acute myocardial infarction, unstable angina, stable angina and healthy controls (control). **a**: Paired comparisons of plasma apoCIII levels in subgroups with acute myocardial infarction, unstable angina, stable angina and control; **b**: Paired comparisons of plasma TNF-α levels in subgroups with acute myocardial infarction, unstable angina, stable angina and control; **c**: Paired comparisons of plasma hs-CRP levels in subgroups with acute myocardial infarction, unstable angina, stable angina and control. a: compared to control, *P* < 0.01; b: compared to stable angina, *P* < 0.01; c: compared to unstable angina, *P* < 0.01

Plasma apoCIII, hs-CRP and TNF-α levels in the CHD subgroups with mild, moderate, and severe stenosis were enhanced compared to those in the healthy control group (*P* < 0.01), while there were no significant differences between each pair of subgroups with severe, moderate, and mild stenosis (*P* > 0.05) (Fig. 2). Plasma apoCIII, hs-CRP and TNF-α levels in the CHD subgroups with AMI, unstable angina, and stable angina were increased compared to those in the healthy control group (*P* < 0.01), with significant differences also identified between each pair of subgroups with AMI, unstable angina, and stable angina (*P* < 0.01) (Fig. 3). The highest apoCIII, hs-CRP and TNF-α levels were detected in the CHD patient subgroups with AMI, followed by those with unstable angina and then those with stable angina.

### Multiple unconditional logistic regression analysis of the effects of plasma apoCIII, hs-CRP and TNF-α levels on the risk of coronary heart diseases

In multiple unconditional logistic regression analysis, coronary heart disease was used as the dependent variable (CHD: yes = 1, no = 0), while the independent variables were defined according to the median: plasma TNF-α (< 12.15 pg/mL =0, ≥12.15 pg/mL =1), plasma hs-CRP (< 3.10 mg/L = 0, ≥3.10 mg/L = 1), plasma apoCIII (< 13.11 mg/dL =0, ≥13.11 mg/dL =1). According to the results, plasma apoCIII, hs-CRP and TNF-α contributed the development of CHD (OR = 2.554, 6.035, 7.252, *P* < 0.01) (Table 5).

**Table 5:** Multiple unconditional logistic regression analysis of the effects of plasma apoCIII, hs-CRP and TNF-α levels on the risk of coronary heart diseases

Parameter		B	*P*	OR	95% CI for OR	
					Lower	Upper
TNF-α (pg/mL)	< 12.15 = 0, ≥12.15 = 1	1.981	0.00	7.252	4.385	11.993
Hs-CRP(mg/L)	< 3.10 = 0, ≥3.10 = 1	1.798	0.00	6.035	3.690	9.871
ApoCIII (mg/dL)	< 13.11 = 0, ≥13.11 = 1	0.938	0.00	2.554	1.547	4.217

*ApoCIII* Apolipoprotein CIII, *TNF-α* Tumor necrosis factor α, *Hs-CRP* high-sensitivity C-reactive protein. Multiple unconditional logistic regressions

### Paired associations of plasma apoCIII, hs-CRP and TNF-α in CHD patients

**Fig. 4:**
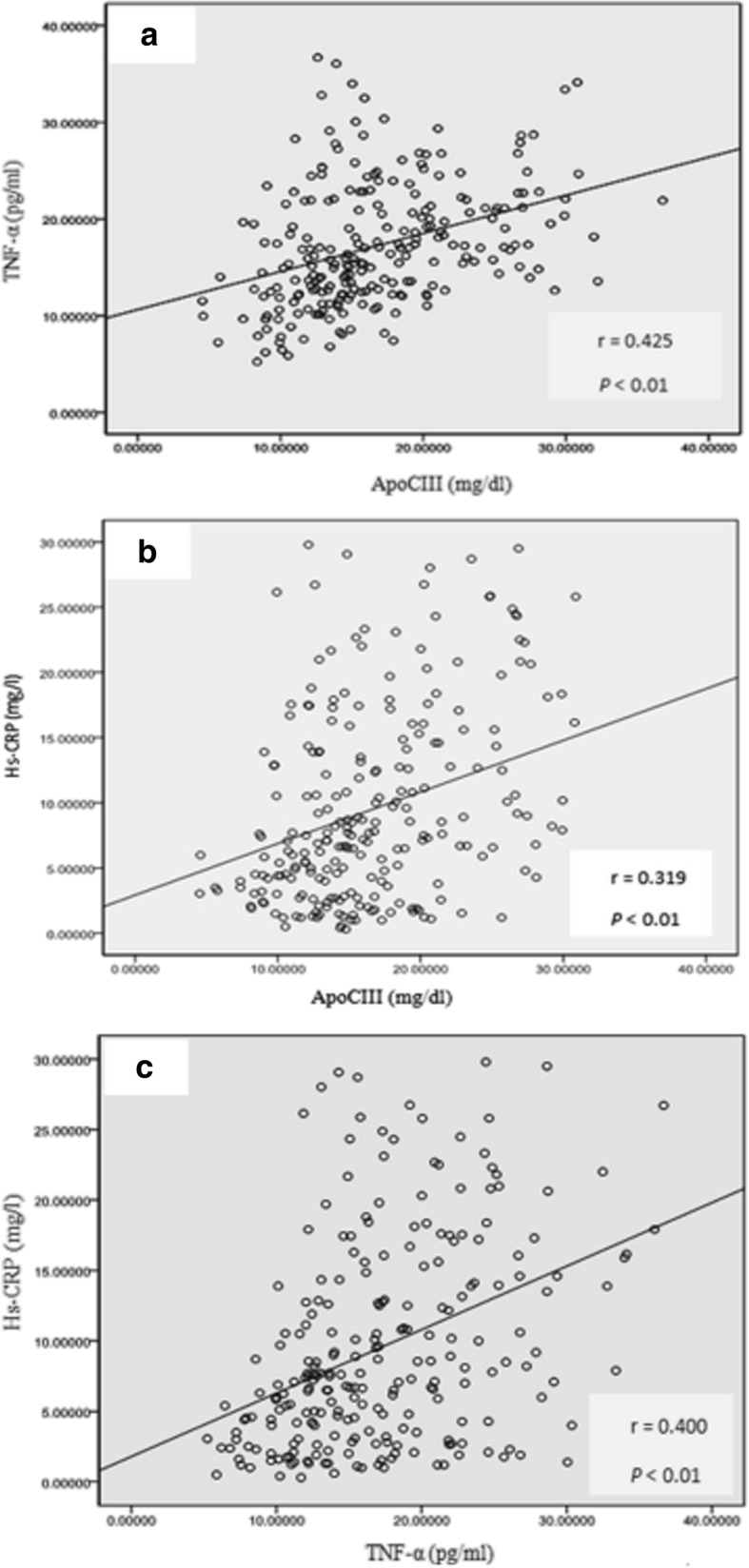
Paired associations of plasma apoCIII, TNF-α and hs-CRP in patients with coronary heart disease. **a** Association of plasma apoCIII and TNF-α in patients with coronary heart disease; **b** Association of plasma apoCIII and hs-CRP in patients with coronary heart disease; **c** Association of plasma TNF-α and hs-CRP in patients with coronary heart disease

Plasma apoCIII was correlated with hs-CRP and TNF-α (*r* = 0.425, *r* = 0.319; *P* < 0.01), and hs-CRP was correlated with TNF-α (*r* = 0.400, *P* < 0.01) in CHD patients (Fig. 4).

## Discussion

ApoCIII has been confirmed to decrease lipoprotein lipase (LPL) activity and result in hypertriglyceridemia, which is an independent risk factor for atherosclerosis [13, 14]. Inflammation facilitates the formation of atherosclerotic lesions and atherosclerotic plaque instability. ApoCIII is also known to upregulate expression of vascular cell adhesion molecule-1 (VCAM-1) and intercellular cell adhesion molecule-1 (ICAM-1) in endothelial cells, and has been shown to induce adhesion of THP-1 cells to EC [2, 3]. ApoCIII is associated not only with dyslipidemia, but also inflammation, both of which play important roles in the development of CHD.

Our study confirmed previous reports of the existence of dyslipidemia (enhanced TG, TC, LDL-C, apoB, Lp(a), apoCIII and lower HDL-C and apoAI) and inflammation (increased TNF-α and hs-CRP) in CHD patients [15, 16].

Inflammation plays a crucial role in the stability of atherosclerotic plaques. In this study, there was no significant variation in the inflammatory markers (TNF-α and hs-CRP) in the subgroups of CHD with severe, moderate, and mild stenosis, while there were marked differences between each pair of subgroups of CHD patients with AMI, unstable angina and stable angina. Furthermore, the association of apoCIII, TNF-α and hs-CRP was also confirmed in CHD patients. CHD is caused by stenosis of the coronary artery, although the clinical features and prognosis are mainly dependent on the instability of atherosclerotic plaques, rather than the extent of stenosis. Our study reveals the role of apoCIII in inflammation and CHD, a finding that is also supported at the cellular level [2, 7].

In addition to the plasma levels, the distribution of apoCIII also plays crucial roles in development of CHD. A previous report indicated that plasma apoCIII was not increased in CHD patients, while, apoCIII in HDL (HDL-apoCIII) was significantly higher and apoCIII in VLDL (VLDL-apoCIII) was greatly reduced in CHD patients [5, 17]. ApoCIII enrichment in HDL could impair HDL-mediated cholesterol efflux capacity, and compromise the anti-atherogenic effect of HDL [18]. ApoCIII in HDL, VLDL and LDL is associated with increased risk of CHD [19, 20]. Statins have been shown to abolish the association of apoCIII with plasma TG [5] in CHD patients and ameliorate apoCIII-induced inflammation [21], which protect against the progression of CHD.

Diet also affects lipid profiles, and it can be speculated that people on a high lipid diet will exhibit unfavorable lipid profiles, while those maintained on nutraceutical and functional food might exhibit more favorable lipid profiles. Nutraceutical and functional foods improve unfavorable lipid profiles when statins are unavailable or as an adjunct to a standardized pharmacological treatment for dislipedimia and CHD [22].

In our study, we obtained information on diet as well as smoking and alcohol status by using a standardized questionnaire. Therefore, it should be noted that obtaining this information may be limited by participant compliance or by the educational status of participants, which may affect their ability to understand the questions.

## Conclusion

The results of our study indicate that the associations of plasma apoCIII, hs-CRP and TNF-α interact with unfavorable lipid profiles to contribute to the clinical features of CHD with stable angina, unstable angina, and AMI in both the Li and Han ethnic groups in China.

## Abbreviations

ApoAI: Lipoprotein AI
ApoB: Lipoprotein B
ApoCIII: Apolipoprotein CIII
CHD: Coronary heart disease
EC: Endothelial cell
EDTA: Ethylene diamine tetra-acetic acid
ELISA: Enzyme-linked immunosorbent assay
FBS: Fasting blood sugar
HDL: High density lipoprotein
HDL-C: High density lipoprotein cholesterol
Hs-CRP: High-sensitivity C-reactive protein
ICAM-1: Intercellular cell adhesion molecule-1
LDL: Low density lipoprotein
LDL-C: Low density lipoprotein cholesterol
Lp(a): Lipoprotein a
LPL: Lipoprotein lipase
Lp-PLA_2_: Lipoprotein-associated phospholipase A2
TC: Total cholesterol
TG: Triglyceride
THP-1: Human acute monocytic leukemia cell line
TNF-α: Tumor necrosis factor α
VCAM-1: Vascular cell adhesion molecule-1
VLDL: Very low density lipoprotein

## References

[1] Zheng C, Khoo C, Furtado J, Sacks FM (2012). Apolipoprotien C-III and the metabolic basis for hypertriglyceridemia and the dense low-density lipoprotein phenotype. Circulation.

[2] Kawakami A, Aikawa M, Alcaide P, Luscinskas FW, Libby P, Sacks FM (2006). Apolipoprotien CIII induces expression of vascular cell adhesion molecule-1 in vascular endothelial cells and increases adhesion of monocytic cells. Circulation.

[3] Kawakami A, Aikawa M, Libby P, Alcaide P, Luscinskas FW, Sacks FM (2006). Apolipoprotien CIII in apoliprotein B lipoproteins enhances the adhesion of human monocytic cells to endothelial cells. Circulation.

[4] Kawakami A, Aikawa M, Nitta N, Yoshida M, Libby P, Sacks FM (2007). Apolipoprotein CIII–induced THP-1 cell adhesion to endothelial cells involves pertussis toxin–sensitive G protein and protein kinase Cα–mediated nuclear factor-ĸB activation. Arterioscler Thromb Vasc Biol.

[5] Xiong X, Liu H, Hua L, Zhao H, Wang D, Li Y (2015). The association of HDL-apoCIII with coronary heart disease and the effect of statin treatment on it. Lipids Health Dis.

[6] Sun M, Chen L, Liu H, Ma L, Wang T, Liu Y (2017). Association of the S2 allele of the SstI polymorphism in the apoC3 gene with plasma apoCIII interacts with unfavorable lipid profiles to contribute to atherosclerosis in the li ethnic group in China. Lipids Health Dis.

[7] Han X, Wang T, Zhang J, Liu X, Li Z, Wang G, Song Q, Pang D, Ouyang H, Tang X (2015). Apolipoprotein CIII regulates lipoprotein-associated phospholipase A_2_ expression via the MAPK and NF-ĸB pathways. Biol Open.

[8] Rosenson RS, Stafforini DM (2012). Modulation of oxidative stress, inflammation, and atherosclerosis by lipoprotein-associated phospholipase A2. J Lipid Res.

[9] Lavi S, McConnell JP, Rihal CS, Prasad A, Mathew V, Lerman LO, Lerman A (2007). Local production of lipoprotein-associated phospholipase A2 and lysophosphatidylcholine in the coronary circulation: association with early coronary atherosclerosis and endothelial dysfunction in humans. Circulation.

[10] Paiva AA, Raposo HF, Wanschel AC, Nardelli TR, Oliveira HC (2017). Apolipoprotein CIII overexpression-induced hypertriglyceridemia increases nonalcoholic fatty liver disease in association with inflammation and cell death. Oxidative Med Cell Longev.

[11] Thygesen K, Alpert JS, Jaffe AS, Simoons ML, Chaitman BR, White HD (2012). Third universal definition of myocardial infarction. J Am Coll Cardiol.

[12] Sullivan DR, Marwick TH, Freedman SB (1990). A new method of scoring coronary angiograms to reflect extent of coronary atherosclerosis and improve correlation with major risk factors. Am Heart J.

[13] Ding Y, Wang Y, Zhu H, Fan J, Yu L, Liu G, Liu E (2011). Hypertriglyceridemia and delayed clearance of fat load in transgenic rabbits expressing human apolipoprotein CIII. Transgenic Res.

[14] Wei J, Ouyang H, Wang Y, Pang D, Cong NX, Wang T, Leng B, Li D, Li X, Wu R (2012). Characterization of a hypertriglyceridemic transgenic miniature pig model expressing human apolipoprotein CIII. FEBS J.

[15] Yan LR, Wang DX, Liu H, Zhang XX, Zhao H, Hua L, Xu P, Li YS (2014). A pro-Atherogenic HDL profile in coronary heart disease patients: an iTRAQ labelling-based proteomic approach. PLoS One.

[16] Chang PY, Lee CM, Hsu HC, Lin HJ, Chien KL, Chen MF, Chen CH, Lee YT, Yang CY (2012). Identification of the HDL-ApoCIII to VLDL-ApoCIII ratio as a predictor of coronary artery disease in the general population: the chin-Shan community cardiovascular cohort (CCCC) study in Taiwan. Lipids Health Dis.

[17] Wyler von Ballmoos MC, Haring B, Sacks FM (2015). The risk of cardiovascular events with increased apolipoprotein CIII: a systematic review and meta-analysis. J Clin Lipidol.

[18] Luo M, Liu A, Wang S, Wang T, Hu D, Wu S, Peng D (2017). ApoCIII enrichment in HDL impairs HDL-mediated cholesterol efflux capacity. Sci Rep.

[19] Jensen MK, Rimm EB, Furtado JD, Sacks FM (2012). Apolipoprotein C-III as a Potential Modulator of the Association Between HDL-Cholesterol and Incident Coronary Heart Disease. J Am Heart Assoc.

[20] Mendivil CO, Rimm EB, Furtado J, Chiuve SE, Sacks FM (2011). Low-density lipoproteins containing apolipoprotein C-III and the risk of coronary heart disease. Circulation.

[21] Zheng C, Azcutia V, Aikawa E, Figueiredo JL, Croce K, Sonoki H, Sacks FM, Luscinskas FW, Aikawa M (2013). Statins suppress apolipoprotein CIII-induced vascular endothelial cell activation and monocyte adhesion. Eur Heart J.

[22] Scicchitano P, Cameli M, Maiello M, Modesti PA, Muiesan ML, Novo S, Palmiero P, Saba PS, Pedrinelli R, Ciccone MM (2014). Nutraceuticals and dyslipideamia: beyond the common therapeutics. J Funct Foods.

